# We Need a “Keeling Curve” Approach for Contaminants of Emerging Concern

**DOI:** 10.1021/acs.est.3c03813

**Published:** 2023-07-07

**Authors:** Adam W. Cooper, Mickey M. Rogers, Kara J. Wiggin, Jonathan H. Slade

**Affiliations:** † Department of Chemistry & Biochemistry, University of California, San Diego, La Jolla, California 92093, United States; ‡ Environmental Molecular Sciences Laboratory, Pacific Northwest National Laboratory, Richland, Washington 99354, United States; § Scripps Institution of Oceanography, University of California, San Diego, La Jolla, California 92093, United States

**Keywords:** environmental pollution, contamination, air pollution, microplastics, policy

Several chemical and particulate species have been designated as contaminants of emerging concern (CECs) due to their persistence in the environment, their detrimental effects on human health and ecosystems, and their lack of current regulations. According to the definitions provided by the U.S. Environmental Protection Agency (EPA) and the United Nations Environmental Programme (UNEP), CECs encompass various substances, including industrial additives such as per- and polyfluoroalkyl substances (PFAS), pharmaceuticals and personal care products (PPCPs), and microplastics.^1^ These CECs have been detected around the globe in previously pristine environments that were once considered untouched by human influence, such as remote high-elevation mountain areas, Arctic air, snowpack, and the open ocean.^1,2^ Similar to greenhouse gases, CECs pose a pervasive threat to all regions of the world.

Monitoring the levels of CECs in the air, land, and water is challenging due to the lack of a standardized methodology for collection and analysis and uncertainty surrounding the effectiveness of regulations. Thus, a crucial question is raised. Is the current approach, which relies on nonstandardized measurements and reporting of CECs, effective in gaining widespread public support for reduction targets? Without clear indications that such policies are leading to reduced levels of CECs, we must consider the need for standardization. In this work, we contend that the standardization of methodologies and consistent reporting of CEC concentrations over time, both at a single reference measurement site and across different environmental compartments, will be essential in comprehending the science behind CECs in the environment and guiding future policy decisions.

The challenges of method standardization and policy action to reduce CEC concentrations can be likened to the historical challenge of measuring and reporting global atmospheric concentrations of carbon dioxide (CO_2_), a greenhouse gas, before the establishment of the renowned Keeling curve in the 1950s.^3^ This curve graphically displays the long-term increase and seasonal variations of CO_2_ in the atmosphere. Prior to the Keeling curve, measurements of CO_2_ were conducted inconsistently using various analytical techniques and locations, resulting in less reliable data for monitoring global averages and trends over time. The creation of the curve and its effectiveness in promoting international collaboration on climate policy were based on two fundamental principles: (1) the development and widespread adoption of a single instrument, the gas manometer, and (2) the meticulous selection of a reference measurement site.

We demonstrate how the principles that underpinned the construction of the Keeling curve and its ongoing success in fostering international collaboration can be applied to the monitoring, reporting, and implementation of policies concerning CECs. To draw a parallel, we focus on the pressing issue of airborne microplastic pollution, which serves as a representative CEC found in all environmental compartments. Microplastics are a prominent topic leading up to the anticipated UN Plastics Treaty scheduled for 2024, which aims to establish a legally binding agreement to “End Plastic Pollution”.^4^

Various techniques are currently employed to analyze microplastic particles in the air. Some rely on single-particle counting paired with spectroscopic techniques for identifying plastic chemical signatures; however, these often have a limited size resolution. Conversely, there are promising methods capable of quantifying the constituent polymers of the plastic in nearly real time, with detection capabilities down to the picogram level.^100^ These techniques differ in their physical and chemical resolution and are inconsistently applied across different monitoring sites.

**Figure 1:**
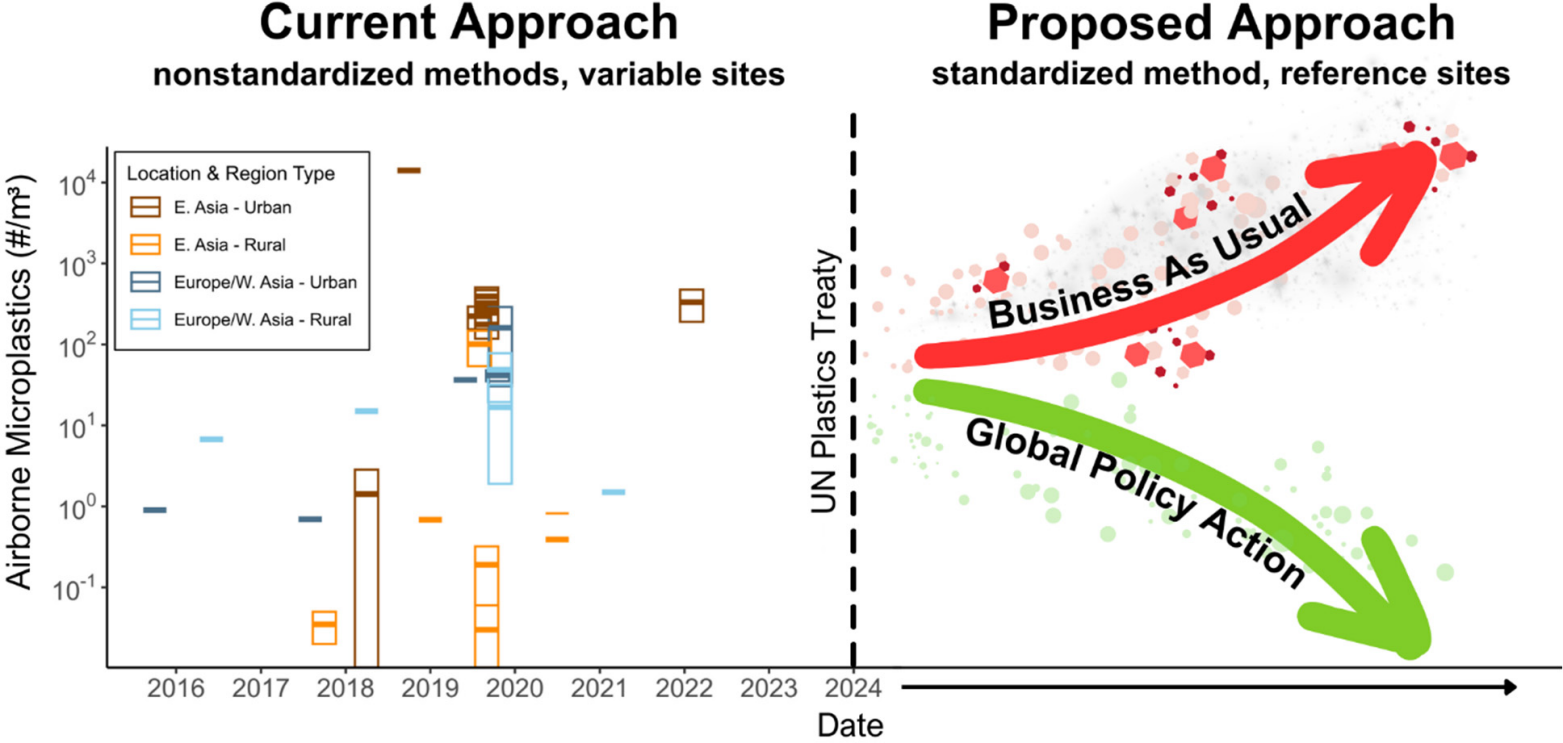
Illustration demonstrating challenges in interpreting global trends of concentrations of contaminants of emerging concern (CECs) based on current monitoring approaches, using airborne microplastics as a representative example. Number concentrations of airborne microplastics over time, as currently analyzed using various methods and measurement sites (left).^5−12^ Anticipated outcomes from using a standardized method at a reference measurement site (right). The red arrow represents an expected upward trend in the concentration of airborne microplastics if the 2024 UN Plastics Treaty is not successful, while the green line represents an expected downward trend if the treaty is successful.

There are limitations to relying on different techniques when quantifying airborne microplastics over time (Figure 1). Due to the lack of consistent measurements, there are no discernible trends over time, and there is significant variability within and between rural and urban locations. Similar graphs can be constructed for other microplastics and CECs measured in different environmental compartments. Thus, implementing a live and continuously updated graph that charts the concentrations of airborne microplastics over time, akin to the iconic Keeling curve for CO_2_, holds significant potential as a powerful tool in addressing the issue of microplastic pollution. This proposed solution, applicable to all CECs, consists of two critical steps that mirror the principles on which the Keeling curve was founded: (1) the adoption of a standardized technique for continuous online monitoring and (2) the identification of a representative measurement site, similar to the Mauna Loa observatory for monitoring CO_2_, and the creation of a global sampling map that acknowledges regional variability. This is crucial as microplastics and other CECs are not as long-lived or as uniformly distributed in the environment as greenhouse gases like CO_2_. One such potential measurement site for airborne microplastics is the Pic du Midi observatory in the pristine French Pyrenees, which experiences the impact of intercontinental transport of airborne microplastics.^13^ Additionally, dedicated networks like the U.S. EPA’s National Air Toxics Trends Sites (NATTS) have been consistently monitoring hazardous air pollutants in the United States since its establishment in 2003. Similar networks and reference sites should be established to monitor CECs in terrestrial, atmospheric, and aquatic environments.

Although there is currently no single technique capable of unambiguously identifying and quantifying all types of CECs in various environmental compartments, it is crucial for the international scientific community to reach a consensus on a standardized technique suitable for specific CECs and matrices. For airborne microplastics, techniques that directly quantify the constituent polymers in a mass-based format, eliminating biases in counting techniques, would reduce instances of human error and particle count inflation caused by plastic breakdown in the environment. Moreover, these techniques would allow for the timely dissemination of a large volume of results. This standardized approach will ensure accurate and verified comparisons between measurements over time and enable the tracking of progress in reducing the levels of CECs in the environment. Such trends can be illustrated to assess the efficacy of policy action, as shown for airborne microplastics in Figure 1.

Persistent environmental CECs, such as plastic, which is widely used in daily life, have the potential to endure for hundreds of years, or even indefinitely.^14^ Given this sobering reality, it becomes crucial to meticulously measure their background concentrations across all environmental compartments in a standardized manner to fully grasp the long-lasting effects of such pollution.

The development of a standardized and easily understandable graph, similar to the Keeling curve, has the potential to serve as a powerful catalyst for global action against CECs. By taking these necessary steps, we can provide a clear and comprehensive road map for stakeholders to track progress toward policy objectives. Such a graph has the potential to inspire international collaboration as an effective response to this pressing issue.
